# A Systems Biology Approach to Memory Health: Integrating Network Pharmacology, Gut Microbiota, and Multi-Omics for Health Functional Foods

**DOI:** 10.3390/ijms26146698

**Published:** 2025-07-12

**Authors:** Heng Yuan, Junyu Zhou, Hongbao Li, Suna Kang, Sunmin Park

**Affiliations:** 1 Department of Physiology and Pathophysiology, School of Basic Medical Sciences, Xi’an Jiaotong University, Xi’an 710049, China; yuanheng.changan@gmail.com (H.Y.); hongbaoli1985@163.com (H.L.); 2 Institute of Advanced Clinical Medicine, Peking University, Beijing 100871, China; zjy888zjy888@gmail.com; 3 Department of Food and Nutrition, Obesity/Diabetes Research Center, Hoseo University, Asan 31499, Republic of Korea; roypower003@naver.com

**Keywords:** health functional foods, memory impairment, biomarkers, gut–brain axis, network pharmacology, multi-omics integration

## Abstract

Memory impairment, ranging from mild memory impairment to neurodegenerative diseases such as Alzheimer’s disease, poses an escalating global health challenge that necessitates multi-targeted interventions to prevent progression. Health functional foods (HFFs), which include bioactive dietary compounds that not only provide basic nutrition but also function beyond that to modulate physiological pathways, offer a promising non-pharmacological strategy to preserve memory function. This review presents an integrative framework for the discovery, evaluation, and clinical translation of biomarkers responsive to HFFs in the context of preventing memory impairment. We examine both established clinical biomarkers, such as amyloid-β and tau in the cerebrospinal fluid, neuroimaging indicators, and memory assessments, as well as emerging nutritionally sensitive markers including cytokines, microRNAs, gut microbiota signatures, epigenetic modifications, and neuroactive metabolites. By leveraging systems biology approaches, we explore how network pharmacology, gut–brain axis modulation, and multi-omics integration can help to elucidate the complex interactions between HFF components and memory-related pathways such as neuroinflammation, oxidative stress, synaptic plasticity, and metabolic regulation. The review also addresses the translational pipeline for HFFs, from formulation and standardization to regulatory frameworks and clinical development, with an emphasis on precision nutrition strategies and cross-disciplinary integration. Ultimately, we propose a paradigm shift in memory health interventions, positioning HFFs as scientifically validated compounds for personalized nutrition within a preventative memory function framework.

## 1. Introduction

Memory impairment represents one of the most significant global health challenges of the 21st century, manifesting across a diverse spectrum of conditions that span from normal aging processes to pathological neurodegenerative diseases. The continuum encompasses age-associated cognitive decline, stress-induced memory dysfunction, post-infectious cognitive symptoms (including long COVID), vascular cognitive impairment, mild cognitive impairment (MCI), and ultimately severe neurodegenerative conditions such as Alzheimer’s disease (AD) [1]. According to the World Health Organization (WHO), over 55 million people currently live with dementia worldwide, with nearly 10 million new cases annually, a figure projected to reach 139 million by 2050. However, these statistics represent only the most severe end of the memory impairment spectrum, as millions more experience subclinical memory decline that significantly impacts the quality of life. The economic burden exceeds $1.3 trillion annually and is anticipated to double by 2030 [1,2].

Clinically, memory impairment encompasses various forms of cognitive decline that can affect individuals across different age groups and circumstances. While some forms represent normative aging processes, others result from modifiable factors such as chronic stress, inflammatory conditions, vascular dysfunction, or infectious diseases [3]. The underlying pathophysiology varies considerably across this spectrum but commonly involves overlapping mechanisms including neuroinflammation, oxidative stress, synaptic dysfunction, mitochondrial impairment, dysregulated neurotrophic signaling, and, in some cases, the accumulation of misfolded proteins [4,5]. The pathophysiological processes result from complex interactions between genetic predispositions and modifiable environmental factors—including diet, physical activity, stress management, and microbiome composition.

Early detection remains a critical challenge across all forms of memory impairment. Age-related cognitive decline may begin in the fourth decade of life, stress-induced memory problems can emerge at any age, post-infectious cognitive symptoms may develop following viral or bacterial infections, and vascular cognitive impairment often progresses silently alongside cardiovascular disease. Each condition presents unique diagnostic challenges, as memory changes frequently begin decades before clinical recognition and may progress along different trajectories. While AD serves as a well-characterized model for severe memory impairment, the integrated systems biology approach is designed to be applicable across the entire spectrum of memory disorders, recognizing that the network pharmacology, gut microbiota, and multi-omics methodologies can identify biomarkers and mechanisms relevant to various forms of memory dysfunction, from normal aging to pathological conditions.

Conventional diagnostic methods such as neuroimaging and cerebrospinal fluid analysis are invasive, expensive, and typically detect changes after substantial neural damage has occurred [6,7]. Moreover, these approaches are often optimized for specific conditions like AD and may not capture the subtle, early changes characteristic of other forms of memory impairment. The diagnostic gap presents particular difficulties in developing clinically validated health functional foods (HFFs), necessitating the identification of non-invasive, cost-effective biomarkers capable of detecting subtle changes across the spectrum of memory function.

HFFs are dietary components that offer physiological benefits beyond basic nutrition and represent promising non-pharmacological interventions to preserve cognitive function across diverse populations and conditions [8]. Bioactive compounds including polyphenols, omega-3 fatty acids, antioxidants, and pre-/probiotics can modulate numerous biological processes implicated in memory decline through mechanisms that may be relevant to multiple forms of cognitive impairment [9]. Despite encouraging findings in preclinical studies, the pathway to clinical application remains underdeveloped due to a lack of validated biomarkers that can assess efficacy across different types of memory impairment, insufficient mechanistic understanding of how interventions affect various pathological processes, and the absence of integrated frameworks connecting molecular pathways to clinical efficacy across diverse populations [10].

The extensive literature review addresses memory impairment across its full spectrum by combining data from three complementary methodologies: network pharmacology, gut microbiota research, and multi-omics approaches. Through this innovative integrated framework, we aim to carry out the following: (1) identify early, modifiable biomarkers of memory function impairment responsive to nutritional intervention across various conditions; (2) validate the biomarker profiles of the HFF in different contexts of memory dysfunction; (3) elucidate the biological pathways through which HFFs exert their effects across the spectrum of memory-related conditions; and (4) propose an evidence-based methodology for the biomarker-guided design of targeted nutritional interventions that can be tailored to different forms of memory impairment. By connecting mechanistic validation with clinical evidence across diverse memory-related conditions, this review establishes a robust validation framework for confirming HFF effectiveness in preserving memory function and preventing cognitive decline across various populations and clinical approaches.

## 2. Biomarkers in Memory Impairment: From Clinical Diagnostics to HFF

Biomarkers serve as critical tools for understanding the pathophysiology of memory impairment, enabling early detection and the development of interventions. CSF and MRI biomarkers differ across various neurodegenerative diseases, including Alzheimer’s, frontotemporal dementia, and Parkinson’s disease. In HFF research, biomarkers identify at-risk populations, evaluate the biological impact of dietary components, and establish mechanistic evidence for clinical efficacy. The section examines biomarkers relevant to cognitive decline, categorizing them either as established clinical biomarkers or emerging biomarkers responsive to nutritional modulation.

Table 1 provides an overall comparison of established clinical biomarkers currently used in memory disorder diagnosis and emerging biomarkers that show promise for modulation through functional food interventions. The comparative framework distinguishes between biomarkers with established clinical utility and those amenable to dietary modulation, highlighting their respective strengths, limitations, and potential for integration in HFF research. Understanding this distinction is crucial for developing evidence-based nutritional interventions that can complement traditional diagnostic approaches while targeting modifiable pathophysiological pathways. This section examines biomarkers relevant to cognitive decline, categorizing them either as established clinical biomarkers or emerging biomarkers responsive to nutritional modulation.

### 2.1. Established Clinical Biomarkers for Memory Impairment

Established biomarkers form the foundation of current diagnostic criteria and are essential for identifying individuals with or at risk of neurodegenerative diseases, such as AD. The biomarkers encompass anatomical changes identified through neuroimaging modalities, cerebrospinal fluid (CSF) indicators, blood-based markers, and cognitive performance assessments [11].

#### 2.1.1. Neuroimaging Biomarkers

Structural magnetic resonance imaging (MRI) detects the hippocampal and medial temporal lobe atrophy, characteristic of AD progression. Functional techniques (fMRI, fluorodeoxyglucose positron emission tomography [FDG-PET]) reveal reduced activity in memory-related networks, particularly the default mode network [11,12,13]. Molecular imaging of amyloid-β (Aβ) and tau PET scans can visualize pathological protein aggregates, confirming the hallmark AD pathology [14].

#### 2.1.2. Cerebrospinal Fluid and Blood-Based Biomarkers

CSF biomarkers provide direct insights into central nervous system (CNS) pathology; reduced Aβ1-42 indicates brain Aβ accumulation, while elevated total tau (t-tau) and phosphorylated tau (p-tau) signify neurofibrillary degeneration. Combined, these form the basis of the Aβ, Tau, and Neurodegeneration (AT(N)) classification system [15,16,17]. Blood-based biomarkers, which offer advantages in terms of accessibility and scalability, are gaining clinical acceptance [18], such as the Aβ1-42/Aβ1-40 ratio, plasma p-tau, and neurofilament light chain reflect Aβ burden, tau pathology, and axonal injury, respectively [19,20]. Additionally, systemic inflammatory markers, such as C-reactive protein (CRP), interleukin-6 (IL-6), and tumor necrosis factor-alpha (TNF-α) reflect the neuroinflammatory component of memory disorders [21,22].

#### 2.1.3. Memory Assessments and Digital Biomarkers

Neuropsychological assessments, including the Mini-Mental State Examination (MMSE), Montreal Cognitive Assessment (MoCA), and Alzheimer’s Disease Assessment Scale-Cognitive Subscale (ADAS-Cog), remain essential tools for evaluating functional cognitive changes [23,24]. Emerging digital tools and wearable technology offer continuous cognitive monitoring in real-world settings [25]. For the identification of HFFs and their development for clinical use, these established biomarkers identify appropriate study populations, confirm underlying pathology, and track long-term cognitive outcomes. While traditional cognitive screening instruments such as the MMSE, MoCA, and ADAS-Cog provide valuable standardized assessments, their primary design for dementia screening and AD-specific pathology may limit their sensitivity to detect subtle cognitive changes responsive to HFF interventions across the broader spectrum of memory disorders. A global cognitive assessment should encompass multiple cognitive domains to capture the nuanced effects of nutritional interventions on cognitive function.

Executive function, encompassing cognitive flexibility, inhibitory control, and working memory, represents a critical domain often affected early in memory disorders and potentially responsive to dietary interventions. The Trail Making Test (TMT-A and TMT-B) provides a well-validated measure of processing speed and cognitive flexibility, while the Stroop Test assesses inhibitory control and selective attention. The Wisconsin Card Sorting Test offers a broad evaluation of abstract reasoning and set-shifting abilities. These measures are particularly relevant for HFF research as executive dysfunction can manifest across various memory disorders, including vascular cognitive impairment and age-related cognitive decline, beyond the traditional AD focus. Memory assessment should extend beyond global cognitive screening to include domain-specific evaluations. The Rey Auditory Verbal Learning Test (RAVLT) provides a detailed assessment of episodic memory encoding, storage, and retrieval, offering sensitivity to early memory changes that may precede clinically apparent cognitive impairment. Wechsler Memory Scale subtests, including Logical Memory and Visual Reproduction, assess both verbal and visual memory systems, providing a thorough evaluation of memory dysfunction patterns that may vary across different memory disorders and respond differentially to specific HFF interventions.

Processing speed deficits represent a common feature across memory disorders and may be particularly sensitive to nutritional interventions targeting vascular and metabolic pathways. The Symbol Digit Modalities Test and Digit Symbol Coding provide robust measures of processing speed and psychomotor function. Attention and working memory assessment through the Attention Network Test and N-back tasks can detect subtle cognitive changes in attention networks that may be modulated by HFF components affecting neurotransmitter systems and neuroplasticity pathways. Visuospatial processing, assessed through the Rey Complex Figure Test and Clock Drawing Test, provides insight into posterior cortical function and may be particularly relevant for detecting cognitive changes in vascular cognitive impairment and mixed dementia. Language function evaluation through the Boston Naming Test and semantic fluency tasks can identify early language changes that may respond to HFF interventions targeting neuroinflammation and synaptic plasticity, particularly in frontotemporal and mixed pathology.

The clinical implications for HFF research are significant, as the integration of these diverse cognitive measures within HFF clinical trials will provide a more nuanced understanding of how nutritional interventions affect different aspects of cognitive function, moving beyond the traditional focus on AD-specific measures to encompass the full spectrum of memory disorders and their associated cognitive profiles.

### 2.2. Emerging Biomarkers Amenable to Modulation

In contrast to conventional clinical indicators, emerging biomarkers offer dynamic insights into the molecular and physiological processes underlying cognitive decline that can be modulated through targeted dietary interventions.

#### 2.2.1. Inflammatory and Oxidative Stress Biomarkers

Peripheral inflammatory markers (IL-1β, IL-6, TNF-α, and CRP) linked to cognitive deterioration can be reduced through anti-inflammatory diets rich in polyphenols, omega-3 fatty acids, and the flavonoids in HFFs [26,27,28,29,30,31,32,33,34]. Oxidative stress biomarkers (F2-isoprostanes, malondialdehyde [MDA], protein carbonyls, 8-hydroxy-2′-deoxyguanosine [8-OHdG]) demonstrate sensitivity to antioxidant-rich functional foods including berries, green tea, and turmeric [35,36,37,38].

#### 2.2.2. Metabolic Dysfunction Biomarkers

Metabolic dysfunction, particularly insulin resistance, is a major contributor to cognitive deterioration [39,40]. Systemic insulin resistance is linked to hippocampal insulin resistance, increased tau phosphorylation, and stimulated Aβ aggregation. Biomarkers, including fasting insulin, homeostatic model assessment for insulin resistance (HOMA-IR), glycated hemoglobin (HbA1c), advanced glycation end-products (AGEs), and Aβ42/Aβ40 ratio, reflect metabolic health status and respond to dietary interventions [41,42]. Functional foods, such as whole grains, legumes, and phytoestrogen-containing plants, and bioactive compounds, such as lutein, zeaxanthin, ferulic acid, and luteolin, can favorably modulate these markers, potentially offering protective effects on brain health [42,43,44,45]. It should be noted that the majority of evidence in this section derives from preclinical animal studies, particularly rodent models. While these studies provide valuable mechanistic insights into metabolic dysfunction biomarkers, translation to human populations requires careful consideration of species differences in metabolism, gut microbiota composition, and cognitive assessment methodologies. Future clinical validation studies are essential to confirm these biomarkers’ relevance in human memory disorders and establish their clinical utility for monitoring dietary interventions.

#### 2.2.3. Neuroplasticity and Neurotrophic Markers

Cognitive decline is associated with reduced synaptic plasticity, as measured by peripheral markers such as brain-derived neurotrophic factor (BDNF), nerve growth factor, neurogranin, and synaptophysin [46]. The neurotrophic factors regulate critical signaling pathways that mediate neuroplasticity, including phosphatidylinositol 3-kinases (PI3K)-Akt-mammalian target of rapamycin (mTOR), mitogen-activated protein kinase (MAPK), extracellular signal-regulated kinases (ERK), and cAMP response element-binding protein (CREB) signaling cascades, which collectively support neuronal survival, dendritic branching, and synaptic remodeling [47]. Neuroprotective dietary compounds demonstrate the capacity to modulate these pathways—docosahexaenoic acid (DHA) activates CREB-mediated BDNF expression through G-protein-coupled receptor 40 (GPR40) receptors, curcumin enhances tropomyosin receptor kinase B (TrkB) signaling as a BDNF receptor to stimulate neurotrophic activity and to downstream ERK/MAPK pathway activation [48]. Resveratrol stimulates sirtuin1 (SIRT1)-dependent deacetylation of peroxisome proliferator-activated receptor gamma coactivator-1alpha (PGC-1α), promoting mitochondrial biogenesis and BDNF synthesis [49]. Additionally, flavonoids like epicatechin and quercetin upregulate Wnt signaling pathways, which are crucial for adult hippocampal neurogenesis and synaptic maintenance, providing multiple mechanistic links between nutrition and cognitive resilience [50,51,52].

#### 2.2.4. Gut–Brain Axis Biomarkers

The biomarkers of intestinal permeability and microbiome function reflect gut integrity and neuroinflammatory risk. The levels of zonulin and lipopolysaccharide-binding proteins indicate intestinal barrier function, while microbiota composition and levels of metabolites, including short-chain fatty acids (SCFAs) and kynurenine pathway intermediates, reflect gut-mediated influences on brain function [53]. The biomarkers respond to prebiotics, probiotics, and fermented foods [54,55].

Human studies have demonstrated the clinical relevance of gut–brain axis biomarkers in memory health. A randomized controlled trial in elderly adults showed that probiotic supplementation significantly reduced serum zonulin levels (a biomarker of leaky gut) and improved cognitive performance scores over 12 weeks in a meta-analysis of randomized clinical trials [56,57]. Clinical investigations have revealed that individuals with mild cognitive impairment exhibit altered SCFA profiles, particularly reduced butyrate and propionate levels, compared to cognitively healthy controls [58]. Furthermore, human intervention studies with prebiotic-rich foods have shown measurable improvements in both gut microbiota diversity and cognitive assessment scores, with parallel changes in inflammatory markers such as IL-6 and TNF-α [59,60]. Clinical studies of probiotics in aging populations have established associations between microbiome composition, specifically *Bifidobacterium* and *Lactobacillus* abundance, and performance on memory-related cognitive tasks [61,62,63].

#### 2.2.5. Epigenetic and Genetic Biomarkers

Epigenetic biomarkers (DNA methylation patterns, histone modifications, and chromatin accessibility) reveal how dietary components reprogram cognition-related gene expression [64]. Methyl donors, polyphenols, and butyrate influence these modifications [39,40]. MicroRNA profiles such as miR-132 and miR-146a respond to dietary modulation and affect genes involved in inflammation and synaptic function [65,66]. Polygenic risk scores (PRS) aggregating variants in genes such as apolipoprotein E (*APOE*), *BDNF*, catechol-O-methyltransferase (*COMT*), and presenilin 1 (*PSEN1*) enable personalized risk profiling and precision nutrition strategies [67,68]. When integrated with other biomarkers, PRS can guide precision nutrition strategies, ensuring that HFFs are tailored to an individual’s genetic susceptibility profile [69,70].

Clinical studies have validated the utility of epigenetic and genetic biomarkers in human populations. A longitudinal study of adults aged 50–70 years demonstrated that dietary folate and B-vitamin intake correlated with DNA methylation patterns in genes associated with cognitive function, including NUDT15 and TXNRD1 [71]. Clinical validation studies have confirmed that APOE ε4 carriers show differential responses to omega-3 fatty acid supplementation, with non-carriers demonstrating greater cognitive benefits and corresponding changes in histone modification patterns [72,73]. Population-based studies have established that individuals with high polygenic risk scores for cognitive decline show enhanced responsiveness to Mediterranean diet interventions, as measured by both cognitive assessments and epigenetic age acceleration markers [74]. Additionally, clinical trials using personalized nutrition approaches based on genetic profiling have demonstrated superior outcomes in memory preservation compared to standardized interventions [75,76].

### 2.3. Critical Evaluation of Current Biomarker Limitations and Integrative Approaches for HFF Validation

Despite significant advances in biomarker discovery, several fundamental limitations constrain the effective validation of HFFs for memory preservation. (1) The Biomarker Sensitivity Paradox represents a critical challenge where clinically meaningful cognitive improvements often occur without proportional changes in traditional biomarkers. A 12-week intervention with curcumin showed 28% improvement in working memory tasks but only 8% reduction in plasma Aβ42 levels, questioning whether current biomarkers adequately capture HFF mechanisms [77]. The discrepancy suggests that the biomarker selection may be fundamentally misaligned with the mechanisms through which HFFs exert their beneficial effects. (2) Temporal mismatch limitations further complicate HFF evaluation. While cognitive benefits from omega-3 supplementation emerge within 6–8 weeks, corresponding changes in neuroimaging biomarkers (hippocampal volume, white matter integrity) require 6–12 months to become detectable [78]. The temporal disconnect creates validation gaps where beneficial HFF effects may be dismissed due to inappropriate biomarker timing expectations. The field urgently needs faster-response biomarkers that can capture early mechanistic changes before structural alterations become apparent. (3) Inter-individual variability presents another substantial challenge that current validation frameworks inadequately address. The APOE genotype influences biomarker responsiveness to HFF interventions, with APOE4 carriers showing 40–60% reduced BDNF responses to polyphenol supplementation compared to non-carriers [72]. Current biomarker frameworks inadequately account for this genetic heterogeneity, leading to false-negative results in genetically diverse populations. The limitation suggests that population-level biomarker validation may systematically underestimate efficacy in specific genetic subgroups. (4) Cross-study comparability crisis compounds these issues, as different studies employ varying biomarker cutoffs, analytical methods, and timing protocols, making meaningful meta-analyses virtually impossible. For instance, plasma BDNF measurements vary 3-fold between studies due to different collection protocols and assay methods, preventing reliable cross-study comparisons and hindering evidence synthesis for HFF validation.

Moving forward, successful HFF validation requires fundamental shifts in methodology. We propose developing composite biomarker indices that weigh sensitivity and specificity appropriately, establishing genotype-stratified reference ranges to account for genetic diversity, and creating temporal biomarker cascades that match intervention timelines. Artificial intelligence and machine learning approaches offer promising solutions, but these computational methods must explicitly incorporate the identified biases and limitations to avoid perpetuating existing problems. Most critically, the field must transition from single-biomarker validation toward dynamic, multi-modal assessment frameworks that capture the complexity of HFF mechanisms while remaining clinically feasible. These limitations collectively suggest that current HFF validation approaches may be systematically underestimating efficacy, potentially delaying beneficial interventions from reaching populations who could benefit most. By leveraging integrated biomarker data with full awareness of these limitations, HFFs with targeted mechanisms can be identified and validated for clinical use, enabling iterative design, validation, and personalization for diverse populations [79,80], but only when current methodological limitations are explicitly addressed and overcome.

## 3. Systems-Based Framework for Biomarker Discovery and Functional Food Design

**Figure 1:**
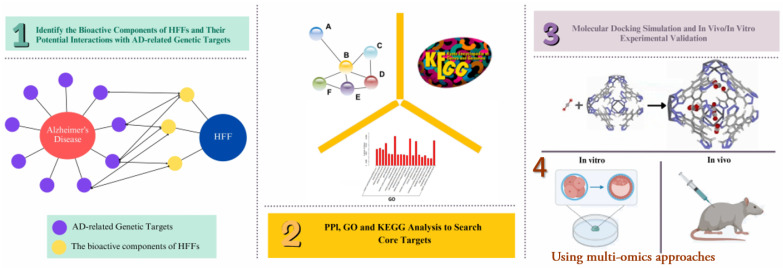
Integrated framework for identifying and validating bioactive components in Health Functional Foods (HFFs) for memory disorder prevention and management. The figure illustrates the systematic approach for evaluating bioactive components in HFFs and their therapeutic potential across multiple memory disorders, including Alzheimer’s disease (AD), mild cognitive impairment (MCI), vascular cognitive impairment, and age-related cognitive decline. The framework integrates three complementary analytical approaches that share the common goal of understanding complex biological systems but differ in scope and methodology, encompassing four integrated components: (1) Network pharmacology analysis demonstrating interactions between bioactive components of HFFs (yellow nodes) and memory disorder-related genetic targets (purple nodes), identified through complete database screening and literature mining. (2) Systems biology analysis incorporating protein–protein interaction (PPI) networks, Gene Ontology (GO), and KEGG pathway enrichment, integrating multi-omics data to identify core therapeutic targets. (3) Molecular docking simulation predicting binding affinity and specificity. (4) Experimental validation through in vitro neuronal cell studies and in vivo animal models, incorporating gut microbiota profiling and multi-omics endpoint measurements over 3–12-month intervention periods. The integrated approach applies to the predictive power of network pharmacology, the mechanistic insights from gut microbiota analysis, and the extensive molecular characterization of multi-omics to ensure robust evaluation of HFF efficacy for memory disorders.

Addressing memory impairment through HFFs requires a paradigm shift from reductionist, single-target approaches to system-level strategies that reflect the multifactorial nature of memory decline. This section outlines an integrated framework that systematically uses three complementary analytical approaches to identify and validate nutritionally modifiable biomarkers for precision memory health interventions (Figure 1).

Network pharmacology serves as the computational foundation, providing rapid predictions of compound-target interactions through computational modeling that focuses on direct molecular binding and pathway modulation. Gut microbiota analysis, integrated throughout the framework, examines indirect effects through microbial metabolism and gut–brain axis signaling, revealing dynamic ecosystem changes over weeks to months and emphasizing host–microbe interactions and metabolite production. Multi-omics approaches combine genomics, proteomics, metabolomics, and transcriptomics data to capture real-time molecular responses across multiple biological layers, providing molecular profiling of actual biological responses.

The three approaches share the common goal of understanding complex biological systems but differ fundamentally in their scope and methodology. While network pharmacology predicts theoretical molecular interactions, gut microbiota analysis reveals ecosystem-level physiological changes, and multi-omics approaches capture overall biological responses across temporal and molecular dimensions. The integrated strategy ensures robust identification of biomarkers that are both mechanistically relevant and nutritionally modifiable.

### 3.1. Network Pharmacology as the Computational Foundation

Network pharmacology serves as the cornerstone of the integrated framework, providing the computational infrastructure necessary to navigate the complexity of HFF-memory health interactions. First proposed by Hopkins in 2007 [81], this approach reconceptualizes bioactive compound effects by modeling drug–target–disease relationships as interconnected networks rather than isolated pathways. In memory health research, this systems-level perspective is crucial because cognitive function emerges from coordinated activity across multiple brain regions, neurotransmitter systems, and molecular pathways disrupted through diverse mechanisms.

#### 3.1.1. Strategic Role Within the Integrated Framework

Network pharmacology fulfills three critical functions that directly support subsequent gut microbiota and multi-omics analyses. (1) Biomarker prioritization engine: Network analysis identifies central hub proteins and pathway nodes most likely to serve as sensitive biomarkers responsive to nutritional intervention. By analyzing the topological properties of compound-target networks, it transforms the vast biomarker landscape into focused candidates for experimental validation, dramatically reducing the burden on subsequent studies. (2) Mechanistic hypothesis generation: Network analysis generates specific, testable hypotheses about HFF mechanisms that directly inform gut microbiota and multi-omics experimental design. If network analysis predicts polyphenol effects through neuroinflammatory pathways, this guides the selection of inflammatory biomarkers for microbiome and multi-omics studies. (3) Cross-methodology integration platform: The network framework enables integration of findings across all three methodologies. Gut microbiota-derived metabolites map onto the same target networks as direct HFF compounds, while multi-omics data overlay onto network structures to validate predicted interactions and identify emergent properties.

#### 3.1.2. Methodological Workflow for Memory Health Applications

The network pharmacology approach employs a systematic pipeline specifically designed for memory disorder biomarker discovery, outlined as follows.

Stage 1: Compound-Target Interaction Analysis. Bioactive food components including flavonoids, anthocyanins, alkaloids, and fatty acids are screened using databases such as TCMSP, PubChem, and SwissTargetPrediction based on pharmacokinetic properties like oral bioavailability (OB) and drug-likeness (DL). It ensures identified targets are nutritionally accessible and neurologically relevant.
Stage 2: Disease-Target Network Construction. Rather than focusing exclusively on Alzheimer’s disease, memory-specific genes linked to diverse impairments are curated from GeneCards, DISGENET, Online Mendelian Inheritance in Man (OMIM), and AlzGene, then intersected with compound targets using visualization tools like Cytoscape (version 3.9.1, 25 March 2025). The broad approach avoids single-condition bias by encompassing age-related decline, vascular cognitive impairment, stress-induced dysfunction, and post-infectious symptoms.
Stage 3: Pathway Enrichment Analysis. Tools such as Database for Annotation, Visualization, and Integrated Discovery (DAVID), Kyoto Encyclopedia of Genes and Genomes (KEGG), and Metascape highlight critical biological pathways amenable to dietary intervention, including PI3K-Akt, nuclear factor kappa-light-chain-enhancer of activated B cells (NF-κB), MAPK, BDNF/TrkB, nuclear factor erythroid 2-related factor 2 (Nrf2), and toll-like receptor signaling. The methodology aids in identifying potential biomarkers such as BDNF, IL-6, tau, and SCFAs for subsequent validation [82,83].

Beyond mechanistic insights, network pharmacology helps precision nutrition through (1) genotype-specific screening, enabling efficacy assessment against wild-type and mutant forms of memory-related proteins such as tau, glycogen synthase kinase 3 beta (GSK-3β), and glyceraldehyde-3-phosphate dehydrogenase (GAPDH); (2) multi-component synergy analysis, particularly valuable for complex extracts where compounds act additively or synergistically; and (3) biomarker prioritization, which refines experimental focus by identifying central nodes most relevant to cognitive health.

#### 3.1.3. Evidence-Based Applications and Validation

Growing research demonstrates these applications across diverse botanical sources. In Coptidis Rhizoma, berberine and ferulic acid modulate memory-related targets like v-rel reticuloendotheliosis viral oncogene homolog A (RELA) and MAPK3, aligning with hypoxia-inducible factor 1 (HIF-1) and PI3K-Akt pathway activity Ye et al. [84]. *Uncaria rhynchophylla*, a plant species, influences 90 AD-relevant targets, including those involved in Aβ and tau regulation, with follow-up studies confirming neuroprotective effects [85]. Eugenol from *Ocimum tenuiflorum* is a GAPDH inhibitor, indicating a link between metabolic-cognitive disease [86]. Similarly, in *Schisandra chinensis*, gomisin A and schisandrin were shown to target prostaglandin-endoperoxide synthase 2 (PTGS2) and acetylcholinesterase (AChE), with studies in animal models validating improvements in neuroinflammation, synaptic function, and metabolic regulation [82].

Curcumin exemplifies the multi-target approach, with network analysis identifying 49 targets including *HMOX1*, *CSF1R*, *NFKB1*, *GSK3B*, and *BACE1*, showing significant expression differences in patients. Its neuroprotective effects involve maintaining cerebral vessel structure, mitochondrial function, and synaptic integrity through anti-amyloid, antioxidant, and anti-inflammatory properties.

For translational impact, computational predictions require experimental validation through in vitro binding assays, cell-based functional studies, in vivo behavioral evaluations, and clinical trials. The tiered approach ensures predictions lead to evidence-based dietary interventions. Future advancements include modeling temporal network dynamics, applying machine learning for enhanced predictions, and designing functional foods tailored to individual genetic risk profiles and biomarker signatures [80,87].

#### 3.1.4. Integration with Gut Microbiota and Multi-Omics

The network pharmacology platform provides computational infrastructure enabling meaningful integration: gut microbiota metabolites map onto target networks alongside direct HFF compounds, revealing indirect gut–brain mechanisms; multi-omics data overlay onto networks to validate predictions and quantify pathway perturbations; and biomarker panel optimization identifies minimal sets providing maximum information about memory health and intervention response. The network pharmacology foundation transforms functional food development from empirical approaches into systematic, predictive science capable of identifying and optimizing biomarkers for precision memory health interventions across diverse populations and clinical approaches.

### 3.2. Gut Microbiota and the Gut–Brain Axis

The gut–brain axis represents a complex bidirectional communication network linking the gastrointestinal tract with the central nervous system through neural, endocrine, immune, and metabolic signaling pathways. Central to this system is the gut microbiota, which influences brain function not only through direct metabolite production but also through the biotransformation of HFF components into bioactive metabolites that can exert profound regulatory effects on memory and cognitive function. In cognitive decline, alterations in gut microbiota composition, microbial metabolite profiles, and gut barrier integrity have been closely associated with neuroinflammation, Aβ deposition, tau hyperphosphorylation, and neuronal cell death [88,89]. Understanding the microbiota’s role as a “metabolic organ” that converts dietary compounds into neuroactive metabolites is crucial for developing precision HFF interventions aimed at preventing memory impairment [90].

Research has revealed significant inter-individual variability in the metabolism of dietary polyphenols, particularly isoflavones and ellagitannins. Isoflavones are metabolized to equol and O-desmethylangolensin, while ellagitannins are converted to urolithins by gut microbiota. The metabolic capacity varies among individuals, resulting in different metabotypes that can be classified as high/low excretors or producers/non-producers. Factors influencing this variability include gut microbiota composition, genetic polymorphisms, age, sex, and BMI. Importantly, these metabotypes have been associated with cardiometabolic risk factors. The urolithin metabotype B in overweight/obese individuals correlates with increased cardiometabolic risk, while urolithin-A production may offer protection against such risks. The findings highlight the potential of polyphenol metabolites as biomarkers for cardiometabolic health and the importance of considering individual variability in metabolic capacity when assessing their effects.

#### 3.2.1. Microbiome Composition and Memory-Related Signatures

Numerous studies demonstrate that the bioactive components in HFFs can beneficially alter gut microbial composition, with direct implications for neuroprotection. Extracts of ginseng, *Forsythia*, and *Hericium erinaceus* and icariin compounds have been shown to restore microbial balance and reduce cognitive deficits in AD models. Protein from ginseng upregulated hippocampal BDNF expression and reduced Aβ deposition, effects that were transferable through fecal transplantation, confirming a microbiota-mediated mechanism [91]. Similarly, *Hericium erinaceus* extract restored redox balance and cognitive performance via gut microbiota modulation and Nrf2 activation [92]. The shifts to beneficial microbes typically mean a reduction in pro-inflammatory genera such as *Helicobacter*, *Desulfovibrio*, and *Allobaculum*, and an increase in the abundance of *Ruminococcus, Akkermansia muciniphila*, and *Anaerotruncus*. The changes in microbial composition correlate with reductions in gut and systemic inflammation, supporting immune homeostasis, blood–brain barrier integrity, and cognitive resilience.

#### 3.2.2. Host–Microbiome–Genotype Interactions and Precision HFF Strategies

Host genetic variation significantly influences the structure and function of the gut microbiome, which in turn affects responsiveness to functional food interventions. Studies have shown that genes regulating mucus production, immune tolerance, and bile acid metabolism affect microbial colonization patterns. Twin and family studies have identified heritable bacterial phyla, such as Firmicutes and Bacteroidetes. Microbiome genome-wide association studies (mGWAS) and Mendelian randomization (MR) analyses have linked host genotypes to gut microbial diversity and specific microbes associated with AD risk [93]. Notably, host genes regulating gut barrier integrity, bile acid metabolism, and immune responses influence microbiota resilience and metabolite profiles, shaping how individuals respond to dietary interventions [94]. Additionally, gut microbes can influence host gene expression via epigenetic reprogramming, including changes in DNA methylation, histone modification, and chromatin accessibility [95]. Functional foods can be tailored based on host genotype-microbiota compatibility, enabling precision nutrition strategies. Individuals with a high PRS for AD might benefit more from SCFA-enhancing foods like resistant starch or inulin-rich herbs, depending on the fermentative capacity of their gut microbiota.

#### 3.2.3. Neuroactive Microbial Metabolites: SCFAs, Bile Acids, and Tryptophan Pathways

HFFs can profoundly influence the metabolic output of gut microbiota. Dietary fibers and polysaccharides are fermented into SCFAs—particularly butyrate, propionate, and acetate—which enhance mucosal barrier integrity, modulate immune function, and increase hippocampal BDNF levels [96]. HFFs such as yam, charred hawthorn, mulberry leaves, *Forsythiae Fructus*, *Cassiae Semen*, hawthorn, and coix seed have been shown to promote SCFA production and alleviate gut inflammation and permeability in both neurological and gastrointestinal models [97].

The tryptophan metabolic pathway, a central node in microbiota–brain communication, is also influenced by diet and microbial composition. HFFs such as yam, mulberry leaves, porcine brain enzyme hydrolysates, and inulin-containing roots enhance cognitive performance by modulating neurotransmitter production by gut microbiota [96]. HFFs that enhance neuroprotective tryptophan metabolites including indole-3-propionic acid and serotonin, can improve neurotransmission and reduce neuroinflammation [98]. Microbiota-modulated bile acids act as ligands for Farnesoid X Receptor (*FXR*) and G-protein-coupled receptor (*TGR5*), which influence memory-related inflammation and mitochondrial function [99,100]. Some HFFs, such as berberine and curcumin, alter bile acid pools to favor neuroprotection [101,102]. The metabolites can serve as biomarkers to evaluate the efficacy of microbial interventions and are measurable in plasma or fecal samples.

#### 3.2.4. Gut–Brain Communication via the Vagus Nerve and Hormonal Signals

The vagus nerve is critical in translating gut microbial signals into central nervous system responses [103]. It acts through both afferent and efferent pathways to regulate inflammation (via the cholinergic anti-inflammatory pathway) and stress responses (via the hypothalamic–pituitary–adrenal [HPA] axis). Animal studies have shown that gut-derived Aβ and bacterial amyloids like “curli” can propagate AD pathology via vagus nerve signaling, whereas vagotomy prevents this transmission and the consequent cognitive deficits [104]. However, vagus nerve stimulation has also been shown to prevent and alleviate memory function, and vagotomy exacerbates memory dysfunction [103,105]. Therefore, vagus nerve-associated changes in cognitive function remain controversial.

Functional foods such as geraniol, polysaccharides from *Polygonatum sibiricum*, and *Polygala tenuifolia* have demonstrated efficacy in modulating vagus-mediated signaling, reducing intestinal and brain inflammation, and alleviating Aβ and tau pathology in AD models [106,107]. In parallel, enteroendocrine hormones like glucagon-like peptide-1 (GLP-1), peptide YY (PYY), and ghrelin—which are regulated by microbial metabolites—play key roles in satiety, stress, mood, and memory. HFFs such as high-fiber grains and prebiotic-rich roots, which enhance SCFAs, can increase GLP-1, influencing synaptic plasticity and stress regulation. Porcine-brain enzyme hydrolysates also activate the parasympathetic nervous system to enhance the gut–brain axis, improving memory function in scopolamine-induced amnesia rat models [97]. The hormones are transmitted through vagal afferents and act on hippocampal and hypothalamic circuits, offering the usage for memory support through diet modification.

#### 3.2.5. Gut Barrier Integrity and Systemic Inflammation

Intestinal permeability, often referred to as “leaky gut,” allows for the translocation of bacterial components like LPS, which can activate peripheral and central immune responses. HFFs that restore tight junction integrity by increasing occludin and zonulin regulation reduce systemic inflammation and lower the risk of Aβ aggregation in both the gut and brain. *Akkermansia muciniphila*, a mucin-degrading bacterium promoted by several HFFs, has been shown to support gut barrier function and reduce neuroinflammatory markers in AD models [108]. The barrier protection may also be associated with reductions in tau hyperphosphorylation, Aβ deposition, and neuronal apoptosis.

#### 3.2.6. Neurotransmitter Modulation by Gut Microbiota

The gut microbiota plays a pivotal role in regulating the biosynthesis and availability of key neurotransmitters, including serotonin (5-HT), gamma-aminobutyric acid (GABA), dopamine (DA), and glutamate (Glu). 5-HT plays a crucial role in regulating mood and improving cognitive function. *Bifidobacterium breve* can increase intestinal and serum 5-hydroxytryptophan (5-HTP) levels and prefrontal cortex and hippocampal 5-HT levels. Serum 5-HTP level demonstrates a positive correlation with brain 5-HT, confirming the significance of gut microbiota in improving cognitive function through modulation of brain 5-HT levels [109,110]. GABA is an inhibitory neurotransmitter, while Glu is an excitatory neurotransmitter and GABA precursor; their imbalance disrupts brain homeostasis and potentially influences pathological processes in AD. The gut microbiota can affect GABA and Glu (GABA precursor)/glutamine (Gln) levels in mouse brains, suggesting a potential influence on cerebral Glu-GABA cycling [111]. DA serves essential functions in various physiological processes, including memory, attention, emotion, and affect.

*Bifidobacterium* species can enhance hippocampal 5-HT levels via increased 5-HTP availability [112]. HFFs to promote GABAergic or serotonergic activity—either directly or via microbiome shifts—may help restore the Glu-GABA balance which is critical for learning and memory [113]. Similarly, bacterial genera such as *Lactobacillus*, *Prevotella*, and *Ruminococcus* influence dopaminergic signaling and cognitive functions like motivation, attention, and reward processing [114].

#### 3.2.7. Microbiota-Mediated Conversion of HFF Components

The gut microbiota functions as a sophisticated biotransformation system that converts ingested HFF compounds into metabolites with enhanced bioavailability and cognitive activity compared to their parent compounds. The microbial metabolism represents a critical determinant of HFF efficacy, as many dietary bioactive compounds require microbial transformation to exert their memory-enhancing effects [115].

In key conversion pathways, research has revealed significant inter-individual variability in polyphenol metabolism, creating distinct “metabotypes” that influence cognitive outcomes. Isoflavones undergo microbial conversion to equol and O-desmethylangolensin, with only 25–30% of individuals possessing equol-producing capacity. Equol demonstrates superior neuroprotective properties compared to its precursor daidzein, including enhanced antioxidant activity and improved blood–brain barrier penetration [116]. Similarly, ellagitannins are converted by gut microbiota to urolithins (A, B, C, and D), with urolithin A showing particular promise for cognitive health through mitochondrial enhancement and neuroinflammation reduction. Flavonoid transformations also yield cognitively active metabolites. Anthocyanins are converted to phenolic acids with enhanced stability, while quercetin undergoes ring-fission to produce metabolites like 3,4-dihydroxyphenylacetic acid, which demonstrates improved memory consolidation effects in clinical studies.

For individual metabolic variability, metabolic capacity varies dramatically among individuals based on gut microbiota composition, genetic polymorphisms, age, sex, and BMI, resulting in metabotypes classified as producers/non-producers. Importantly, urolithin metabotype B in overweight individuals correlates with increased cardiometabolic risk and reduced cognitive benefits, while urolithin-A producers demonstrate enhanced memory performance. The variability necessitates personalized approaches to HFF interventions. In prebiotic-mediated effects, beyond direct transformation, certain HFF components function as prebiotics, promoting beneficial bacteria that produce neuroactive metabolites. Inulin and resistant starch enhance Bifidobacterium and Lactobacillus populations, which produce GABA, acetylcholine precursors, and BDNF-enhancing compounds. Biomarkers can implicate the understanding microbial conversion fundamentally alters biomarker strategies. Wide-ranging panels include both parent compounds and microbial metabolites, along with indicators of individual conversion capacity. The temporal dynamics of conversion—with metabolites showing delayed but sustained elevation—require extended sampling protocols and enable personalized dosing strategies based on metabolic capacity.

### 3.3. Integration of Multi-Omics for Functional Food Design to Prevent Memory Impairment

As memory impairment and neurodegenerative conditions like AD arise from complex and multifactorial etiologies, there is a growing demand for integrative frameworks that uncover mechanistic insights and guide precision intervention. Multi-omics integration—including transcriptomics, proteomics, metabolomics, epigenomics (such as microRNAs), and genomics—offers a systems-level platform for mapping how the bioactive components in HFFs modulate pathways relevant to cognitive decline [117]. The tools enable the identification of predictive biomarkers, make population stratification, and support the rational development of multi-targeted dietary interventions.

#### 3.3.1. Transcriptomic Analyses

Transcriptomics enables the understanding of changes in gene expression in response to functional food interventions, particularly in memory-related pathways such as CREB signaling, cholinergic function, and oxidative stress response. Compounds like purslane amide E from *Portulaca oleracea* and total flavonoids from *Gardenia jasminoides* and cinnamon have shown neuroprotective effects by enhancing the transcription of genes related to the CREB and Nrf2 pathways and regulating the targets of the cholinergic system [118]. Purslane amide E has been shown to alleviate oxidative stress and neurotoxicity in AD mouse models by modulating the expression of apoptotic genes [119]. *Gardenia* extracts influence cholinesterase and carbonic anhydrase activity, thereby enhancing memory through transcription factor regulation and protein kinase R-like endoplasmic reticulum kinase (PERK) pathway modulation [120]. Cinnamaldehyde from cinnamon has also been found to preserve mitochondrial dynamics, reduce oxidative damage, and suppress Aβ accumulation [121]. The findings illustrate the utility of transcriptomics in capturing gene-environment interactions and support the integration of expression quantitative trait loci to identify personalized nutritional responses.

#### 3.3.2. Proteomics Approaches

Proteomic profiling further complements transcriptomic data by revealing post-transcriptional regulation and alterations in the protein network. Functional foods such as schisandrin B and dendrobium nobile Lindl alkaloids (DNLA) have shown efficacy in reducing tau hyperphosphorylation and Aβ-induced neurotoxicity by targeting glycogen synthase kinase (GSK)-3β phosphorylation and regulating apoptotic proteins like caspase-3 [122,123]. Schisandrol A improves synaptic function in tau-transgenic *Drosophila* models by modulating mTOR pathway proteins and suppressing advanced glycation end-product receptor signaling [124]. Likewise, DNLA modulates synaptic plasticity-related proteins such as synapsin-1 and postsynaptic density protein 95 (PSD-95), while downregulating inflammatory cytokines (IL-6, TNF-α, and IL-1β) in AD mouse models [123]. Ginkgo biloba extract and ginsenosides from *Panax ginseng* and *Panax notoginseng* have been shown to regulate the PI3K/Akt/mTOR signaling axis [125], inhibit beta-site amyloid precursor protein cleaving enzyme 1 (BACE1), and enhance α-secretase activity, collectively improving cholinergic neurotransmission and synaptic integrity [126]. The findings validate proteomics as a robust tool for understanding the HFF-driven restoration of synaptic function and neuronal health.

#### 3.3.3. Metabolomic Profiling

Metabolomics captures small-molecule changes that reflect genetic, dietary, and environmental interactions. Alterations in polyamines, branched-chain amino acids, phospholipids, and acylcarnitines have been observed in AD and linked to disease progression. Functional foods such as *Cornus officinalis* influence these metabolites through active compounds like polysaccharides and iridoid glycosides, which regulate hippocampal presenilin-1 (PS1), glycogen synthase kinase 3 beta (GSK3β), and BDNF–TrkB signaling, improving synaptic function [127]. Iridoid glycosides reduce Aβ toxicity and tau phosphorylation, and increase synapsin-1, thereby promoting synaptic plasticity [128]. *Zanthoxylum bungeanum* extract, rich in hydroxy-α-sanshool, enhances the CREB/BDNF axis [129], while its polyphenols boost free radical scavenging and antioxidant capacity [19]. Similarly, *Rubus idaeus* extracts (ethyl acetate, chloroform fractions) support cholinergic function and mitigate oxidative stress in aging mice [130]. Its flavonoids modulate the PI3K/Akt pathway, reduce caspase-1 and BCL2-associated X protein (BAX), and promote B-cell lymphoma/leukemia 2 (Bcl-2) expression, thus enhancing neuronal survival [131]. The findings demonstrate how HFFs can shape the metabolic landscape in favor of cognitive protection. Integrating host metabolic genotype (CYP450 variants) enhances the interpretation of dietary-metabolite interactions.

#### 3.3.4. Epigenomics and MicroRNAs

MicroRNAs (miRNAs) as epigenetic regulators are emerging as vital post-transcriptional regulators and biomarkers for AD and MCI. Epigenetic modifications at loci, like ATP-binding cassette subfamily A member 7 (*ABCA7*) and bridging integrator 1 *(BIN1*), have been linked to the AD burden [132]. Expression of miR-128 increases during mid-stage AD and declines in the late-stage disease [133,134], while miR-206 and miR-132 are elevated in MCI and regulate targets like BDNF and sirtuin 1 (SIRT1). Circulating miR-206 is a promising early predictor of MCI-to-AD conversion [135]. A comparative study identified that miR-31, miR-93, miR-143, and miR-146a were significantly reduced in AD patients, while miR-93 and miR-146a increased in MCI when compared with healthy controls [136]. Nutritional components such as polyphenols, SCFAs, and methyl donors modulate miRNA expression, enabling dietary control over gene regulatory networks linked to memory preservation. The miRNAs can serve as diagnostic biomarkers and as mechanistic mediators of HFF action.

#### 3.3.5. Genomics and Nutritional Precision

Genomic insights, particularly from genome-wide association studies (GWAS), contribute to identifying risk alleles and their impact on HFF efficacy. While early-onset AD is driven by rare mutations in amyloid beta precursor protein (*APP*), *PSEN1*, and *PSEN2*, late-onset AD is polygenic, with apolipoprotein E4 (*APOE ε4*) being the most significant risk allele [137,138]. Notably, population-specific variants, including the triggering receptor expressed on myeloid cells 2 (*TREM2, p.H157Y*), SHANK-associated RH domain interactor (*SHARPIN*), and calcium voltage-gated channel subunit alpha1 A (*CACNA1A*), have been identified in East Asian cohorts [139]. Their variants affect the pathways related to inflammation, cholesterol transport, and synaptic pruning, potentially modifying individual responses to HFFs. Moreover, polymorphisms in metabolic enzymes influence the pharmacokinetics and bioavailability of bioactive compounds. Ginsenoside metabolism is influenced by uridine 5′ diphospho (UDP) glucuronosyltransferase (*UGT*) polymorphisms, which affect the efficacy of Panax-based interventions. Integrating genomic data allows for the stratification of individuals into responder categories and the design of genotype-guided functional food formulations.

To synergize these data streams, computational approaches—including machine learning, network pharmacology, and systems biology modeling—are employed to identify predictive biomarker panels and critical molecular targets [80,81,140]. By integrating transcriptomic, proteomic, and metabolomic changes with genotype and phenotype data, researchers can construct multivariate models to assess individual risk profiles and HFF responsiveness. The convergence of omics science and artificial intelligence supports the emergence of precision nutrition, whereby specific dietary interventions are tailored to an individual’s biological makeup to prevent or delay cognitive decline [141]. Therefore, multi-omics integration offers an integrated framework to decode the molecular mechanisms through which functional foods exert neuroprotective effects. By linking gene expressions, protein activity, metabolite shifts, microRNA regulation, and genetic predisposition, this approach accelerates biomarker discovery and facilitates the rational design of personalized dietary strategies to prevent memory impairment. As these technologies become more accessible, multi-omics-guided functional food development will play an increasingly central role in precision brain health.

#### 3.3.6. Cross-Validation Strategies for HFF Memory Studies

The integration of multi-omics data in HFF research for memory function requires strategic cross-validation approaches to ensure reliable and translatable findings. Given the complexity and resource requirements of extensive omics studies, a tiered validation framework is most practical for establishing mechanistic insights and therapeutic efficacy. There are core cross-validation approaches, detailed as follows: (1) Pathway-Level Cross-Validation: The fundamental approach validates transcriptomic findings through corresponding proteomic and metabolomic changes within the same biological pathways. For HFF memory studies, the upregulation of neuroprotective genes such as phosphorylation of CREB and BDNF mRNA expression should correlate with increased protein expression and downstream metabolite production. Pathway enrichment analysis using KEGG, Reactome, and Gene Ontology databases enables systematic verification of concordant biological processes across omics layers, particularly for memory-relevant pathways including cholinergic signaling, synaptic plasticity, and neuroinflammation. (2) Statistical Cross-Validation: Robust statistical validation prevents overfitting and ensures reproducible biomarker identification. For HFF studies, this includes k-fold cross-validation of machine learning models trained on integrated omics datasets, permutation testing to assess pathway enrichment significance, and bootstrap analysis for biomarker stability assessment. Multi-omics factor analysis identifies shared variation patterns across data types while controlling for technical confounders. There are complementary validation approaches as follows: (1) Functional Cross-Validation: It is recommended for efficacy studies. Mechanistic predictions from multi-omics integration are validated through targeted functional assays relevant to memory function. The study includes acetylcholinesterase activity measurements to confirm cholinergic pathway modulation, synaptic protein quantification (PSD-95, synapsin-1) to verify synaptic plasticity changes, and cognitive behavioral testing (Morris water maze, novel object recognition) to demonstrate functional outcomes corresponding to molecular changes. (2) Model System Cross-Validation: It is applied to translational studies. Findings progress from in vitro neuronal cultures to animal models and ultimately human studies. The approach is particularly valuable for establishing dose–response relationships and identifying translational biomarkers that remain consistent across model systems, though resource constraints may limit full implementation in all HFF studies.

HFF memory studies should prioritize pathway-level and statistical cross-validation as foundational requirements, with functional validation strongly recommended when assessing cognitive efficacy. Additional validation approaches can be incorporated based on study objectives, available resources, and translational goals. The flexible framework ensures scientific rigor while maintaining practical feasibility for advancing HFF research in memory health applications.

## 4. Key Mechanistic Pathways and Biomarkers Modulated by HFF

**Figure 2:**
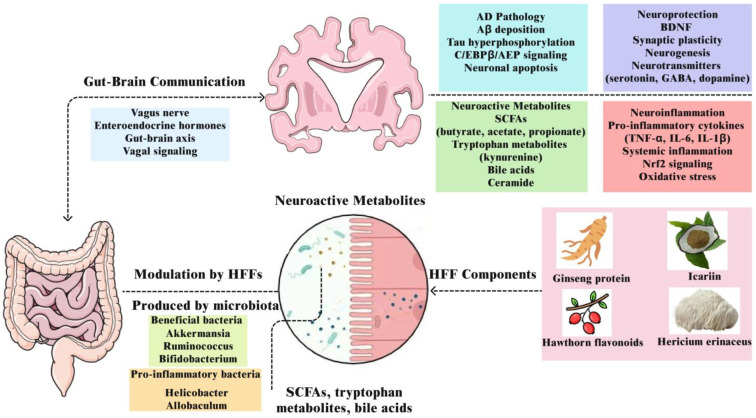
Gut–brain axis communication and Health Functional Food (HFFs) modulation in memory impairment. The schematic illustrates the bidirectional communication between the gut microbiome and brain in memory impairment conditions, including mild cognitive impairment (MCI), Alzheimer’s disease (AD), and ischemia-induced dementia, and potential therapeutic modulation by HFFs. The gut microbiota panel (left) contrasts beneficial bacteria (*Akkermansia*, *Ruminococcus*, *Bifidobacterium*) that support barrier function and reduce inflammation with pro-inflammatory species (*Helicobacter*, *Allobaculum*) associated with increased intestinal permeability in memory impairment disorders. Neuroactive metabolites (center) include short-chain fatty acids (SCFAs), tryptophan derivatives, and bile acids that cross the blood–brain barrier to influence neuroinflammation and cognitive function. The brain pathology section (top) shows four key processes affected in memory impairment: pathological changes (Aβ deposition, tau hyperphosphorylation), neuroprotective mechanisms involving BDNF and synaptic plasticity, metabolite-mediated effects, and neuroinflammatory responses common across MCI, AD, and ischemic conditions. HFF components (right) represent bioactive compounds from various sources that can modulate microbiota composition and brain function. Multi-omics approaches integrate genomic, proteomic, metabolomic, and transcriptomic data to understand HFF mechanisms of action in memory enhancement. Bidirectional communication occurs through multiple pathways: vagal nerve signaling, enteroendocrine hormone release, and direct metabolite transport. Dashed arrows indicate regulatory relationships, while solid arrows show direct communication pathways. The integrated system demonstrates how dietary interventions may influence memory impairment progression through gut–brain axis modulation across different pathological conditions. The dotted lines with arrows: directional flow (implying complex or indirect communication pathways. The dotted lines without arrows: a correlation, association, or a less direct/unknown relationship.

HFFs exert neuroprotective effects through the simultaneous modulation of interconnected biological pathways implicated in memory decline and neurodegeneration, rather than through single mechanisms. Growing evidence demonstrates that bioactive compounds derived from HFF—including polyphenols, saponins, alkaloids, flavonoids, amino acids, peptides, and polysaccharides—can regulate key molecular cascades associated with oxidative stress, neuroinflammation, synaptic plasticity, and gut–brain communication [142]. Understanding these mechanistic targets facilitates the rational development of multi-targeted, biomarker-informed, and personalized dietary interventions. The overall mechanisms underlying memory impairment and therapeutic interventions are illustrated in Figure 2, which depicts the gut–brain axis communication pathways and health functional food modulation. Table 2 provides a detailed summary of experimental studies investigating the molecular mechanisms of herbal extracts, bioactive compounds, and probiotics that target memory enhancement. The studies use multi-omics approaches in animal models to elucidate mechanistic pathways, with findings that offer translational potential for human clinical applications in memory impairment conditions including MCI, AD, and ischemia.

### 4.1. Neuroinflammation and Oxidative Stress: NF-κB, Nrf2, and Cytokines

Neuroinflammation and oxidative stress are two fundamental and interlinked mechanisms underlying the pathogenesis of memory impairment and AD. Oxidative stress arises when the generation of reactive oxygen species (ROS) exceeds the brain’s intrinsic antioxidant defense capacity. Given the brain’s high oxygen demand, abundance of polyunsaturated fatty acids, and low antioxidant reserves, it is particularly vulnerable to ROS-induced damage, which can lead to lipid peroxidation, DNA fragmentation, protein oxidation, and ultimately neuronal death. Aβ aggregation, a hallmark of AD pathology, is both a cause and consequence of oxidative stress. It promotes the formation of superoxide anions, hydrogen peroxide, and reactive aldehydes, such as 4-hydroxynonenal (HNE), further aggravating neuronal injury.

The NF-κB pathway is a key regulator of pro-inflammatory cytokines, including IL-1β, IL-6, and TNF-α, which are frequently elevated in neurodegenerative conditions. Simultaneously, the Nrf2 signaling pathway acts as a master regulator of cellular antioxidant responses, controlling the expression of genes such as heme oxygenase-1 (*HO-1*), nicotinamide adenine dinucleotide phosphate (NAD(P)H), quinone oxidoreductase 1 (NQO1), and glutathione-S-transferases (GSTs). Functional foods rich in bioactive compounds, such as curcumin, resveratrol, berberine, and schisandrin B, suppress NF-κB activation while enhancing Nrf2 translocation and activity.

*Gardenia* extract, *Hericium erinaceus*, and *Coptidis Rhizoma* have demonstrated anti-inflammatory and antioxidant effects in AD models by downregulating *NF-κB* and upregulating *Nrf2*-mediated gene expression [10,11,26]. Additionally, natural tocopherols (vitamin E), abundant in some dietary oils and nuts, are inversely correlated with memory decline in the elderly [145]. The findings suggest that targeting the NF-κB/Nrf2 axis with functional foods may reduce oxidative injury and inflammation in the brain, preserving neuronal function and delaying cognitive deterioration.

### 4.2. Synaptic Plasticity and Neurogenesis: BDNF-TrkB, CREB, Wnt/β-Catenin

Synaptic plasticity—the brain’s ability to reorganize synaptic strength—is essential for memory formation, consolidation, and retrieval. Neurogenesis in the hippocampus, particularly in the dentate gyrus, further contributes to cognitive flexibility and resilience against neurodegenerative damage. Both processes are tightly regulated by molecular pathways such as BDNF-TrkB, CREB phosphorylation, and the Wnt/β-catenin signaling cascade. Disruptions in these pathways are strongly implicated in the pathophysiology of cognitive impairment and AD. The clinical relevance of BDNF pathway modulation is supported by multiple human studies in the elderly, demonstrating direct correlations between serum BDNF levels and cognitive performance [42,146]. The Sefuri study demonstrated that lower brain-derived neurotrophic factor levels are associated with age-related memory impairment in community-dwelling older adults, establishing a clear link between declining BDNF and memory dysfunction in aging populations. Additionally, longitudinal research has shown that serum BDNF levels predict progression to MCI in cognitively normal older adults [42], indicating that BDNF serves as both a biomarker and potential therapeutic target for early intervention. Further clinical evidence from a study of 191 participants (mean age 50.03 ± 2.10 years) revealed that lower plasma BDNF levels were significantly associated with worse memory performance on standardized cognitive assessments, particularly in postmenopausal women, with corresponding alterations in working memory circuitry function observed through fMRI [147]. Clinical evidence establishes BDNF as a critical biomarker linking pathway modulation to measurable cognitive outcomes.

HFFs and their bioactive compounds—such as ginsenosides, icariin, galangin, flavonoids from purslane, and polyphenols from *Zanthoxylum bungeanum*—have demonstrated the capacity to enhance synaptic plasticity in preclinical AD models and also in clinical studies [148]. Purslane amide E improves hippocampal CREB signaling, while ginsenosides stimulate BDNF production and upregulate *TrkB* expression. Furthermore, Wnt/β-catenin activation—important for synapse formation and neurogenesis—is positively regulated by several polyphenols and herbal components, including curcumin, resveratrol, and notoginsenosides, which inhibit GSK-3β, a negative regulator of β-catenin stability. Thus, the restoration of BDNF-CREB-Wnt signaling by functional foods not only prevents synaptic loss but also enhances neuronal regeneration, offering a promising approach to preserving cognitive function.

### 4.3. Gut–Brain Axis and Metabolite Signaling: SCFAs, Tryptophan, Bile Acids

Microbial metabolites such as SCFAs—especially butyrate, propionate, and acetate—are critical in regulating inflammation and maintaining blood–brain barrier (BBB) integrity. Butyrate has demonstrated neuroprotective effects in traumatic brain injury and AD models, improving BBB permeability, enhancing learning-related gene expression, and reducing neuronal loss [149,150]. Functional food ingredients like *Forsythia* extract and geraniol have been shown to increase serum SCFA concentrations while restoring cognitive function in AD animal models [96,151].

Disruptions in tryptophan metabolism, particularly the kynurenine pathway, lead to the accumulation of neurotoxic metabolites that contribute to neurodegeneration. HFFs enriched in prebiotics and polyphenols may modulate tryptophan metabolism to promote serotonergic and anti-inflammatory pathways. Similarly, bile acid metabolism, regulated by gut microbiota and host receptors including FXR and TGR5, influences neuroinflammation and cholesterol turnover, both implicated in AD progression.

Dysbiosis in AD patients is characterized by an increased abundance of pro-inflammatory microbes (*Escherichia coli* and *Shigella*) and a reduced abundance of beneficial species (*Bifidobacterium*, *Butyricicoccus*, and *Clostridium XIVb*) [152,153,154]. Functional foods such as *Polygonatum kingianum* polysaccharides restore microbiota homeostasis, reduce LPS and exotoxin translocation, and lower systemic inflammation, ultimately improving memory outcomes [155]. Taken together, these findings highlight the translational potential of targeting the gut–brain axis through functional foods to reduce neuroinflammation, modulate neuroactive metabolite signaling, and restore cognitive resilience.

## 5. Regulatory Framework and Clinical Development of HFFs for Memory Impairment

### 5.1. Current Regulations for Memory/Cognitive Function Claims in Major Markets

Regulatory frameworks for health claims related to memory function improvements demonstrate significant variation across global markets, with some jurisdictions adopting more progressive approaches than others [156]. The requirements related to health claims for food products vary considerably across South Korea, Japan, Australia, New Zealand, the EU, and the USA. The Ministry of Food and Drug Safety (MFDS) in South Korea has established one of the most complete and health-beneficial regulatory frameworks, recognizing memory improvement as a distinct functional claim category and requiring robust evidence including at least one well-controlled randomized controlled trial (RCT) with validated memory endpoints. The approach helps consumers access scientifically substantiated cognitive health products. In contrast, the European Food Safety Authority (EFSA) maintains a more restrictive stance, primarily approving only claims related to the maintenance of normal brain function while emphasizing mechanistic plausibility and requiring extensive longitudinal data [157]. The U.S. FDA operates under a more limited Qualified Health Claims system, allowing cautiously worded statements linking dietary substances to reduced Alzheimer’s disease risk only when accompanied by appropriate disclaimers [158]. Recent regulatory enforcement, such as the December 2024 FTC ruling prohibiting Prevagen makers from claiming brain function or memory improvement benefits demonstrates the ongoing challenges in the U.S. market. Korea’s progressive regulatory approach thus represents a model that better balances scientific rigor with practical access to beneficial cognitive health interventions.

### 5.2. Evidence Requirements for Claims

Claims related to improvements in memory typically require placebo-controlled RCTs using validated neuropsychological assessment tools, such as Stroop and memory recall tests, demonstrating statistically significant improvements in specific cognitive parameters [159,160]. Prevention claims necessitate epidemiological evidence linking ingredient intake to reduced disease incidence, plus mechanistic studies showing modulation of relevant biological pathways. Current debates center on validating surrogate endpoints such as BDNF, pTau, and Aβ42/40 ratios as biomarkers for efficacy assessment.

### 5.3. Prevention vs. Improvement Claims

The distinction between claims related to prevention and improvement significantly impacts study design and evidence requirements. Improvement claims focus on enhancing the existing cognitive function in the short term, assessed through direct cognitive testing [159,160]. Prevention claims imply risk reduction in cognitive decline, requiring longitudinal cohort studies and evidence of biological plausibility [161,162]. Key regulatory challenges include cross-cultural standardization of cognitive assessment tools and biomarker validation as substitutes for clinical outcomes.

### 5.4. HFFs for Memory Function Available in the Market

Several HFF ingredients with memory enhancement claims have been extensively studied in the scientific literature to establish both efficacy and safety profiles. Efficacy was assessed through in vitro studies, in vivo animal studies, and RCTs. Safety evaluation was conducted either through registration as conventional foods or through animal safety studies. The HFFs had been studied for efficacy and mechanism. Phosphatidylserine has demonstrated particularly robust clinical evidence. Multicenter randomized controlled trials have shown that phosphatidylserine-containing products improve word recall performance in elderly individuals when administered at 300 mg/day. Memory enhancement is further supported by functional magnetic resonance imaging (fMRI) data revealing increased hippocampal activity [143,144,163]. Conversely, the European Food Safety Authority (EFSA) rejected a specific probiotic strain despite promising animal studies, as human trials failed to demonstrate consistent dose–response relationships [164]. Future regulatory frameworks should accommodate multi-modal evidence, including composite biomarker panels, systems biology models, and personalized nutrition approaches. Advancing this field for clinical application requires enhanced collaboration between nutrition scientists, regulatory bodies, and neuroscientists to develop standards balancing innovation with public health responsibility.

### 5.5. Safety Considerations

The safety profile of HFFs for memory disorders represents a critical factor in their clinical development and regulatory approval. While many HFFs originate from traditional foods and herbs with established consumption histories, their concentrated forms and therapeutic applications require careful safety evaluation. The safety evaluation follows a systematic risk assessment approach where HFFs derived from foods or herbs traditionally consumed as water extracts or low-concentration ethanol extracts (≤30%) may not require extensive safety testing due to their established safety profile through historical use. However, this exemption applies specifically to products that maintain traditional preparation methods and concentrations, do not exceed historically consumed dosages, contain no novel processing agents, and have documented safe consumption patterns. For HFFs that deviate from traditional methods, use higher concentrations, or incorporate novel extraction processes, thorough safety testing becomes mandatory.

Several safety considerations are particularly relevant for HFFs targeting memory disorders. Memory disorder patients often take multiple medications including cholinesterase inhibitors, NMDA receptor antagonists, and antipsychotics, requiring careful evaluation of potential pharmacokinetic and pharmacodynamic interactions that could alter drug efficacy or increase adverse effects. Patients with memory disorders may have impaired judgment regarding dosing and increased susceptibility to medication confusion, heightening risks of over- or under-dosing. The elderly population, who predominantly suffer from memory disorders, may have altered metabolism, increased sensitivity to bioactive compounds, and a higher baseline risk for adverse reactions. Additionally, even traditionally safe foods can pose allergenic risks when consumed in concentrated forms.

HFFs occupy a complex regulatory space between traditional foods and pharmaceutical medicines, creating several safety-related challenges. Most countries lack specific regulatory frameworks for HFFs, leading to inconsistent safety standards and oversight. Unlike pharmaceuticals, HFFs may lack standardized manufacturing processes, resulting in batch-to-batch variability in both efficacy and safety profiles. Inadequate quality control can lead to contamination with heavy metals, pesticides, or other harmful substances, while products may contain undeclared ingredients or pharmaceutical adulterants that pose serious safety risks.

To address these safety concerns and support clinical development, several measures are essential including implementing extensive toxicology studies for novel HFF formulations, establishing robust pharmacovigilance systems to monitor adverse events and drug interactions, and developing centralized databases to track safety data across different products. Health technology assessments should evaluate both efficacy and safety profiles, comparing benefit-risk ratios of HFFs against established treatments for memory disorders, while clearer regulatory pathways should ensure appropriate safety oversight while recognizing traditional use history.

The clinical development of HFFs for memory disorders requires a balanced approach that respects traditional use while ensuring modern safety standards through risk-stratified approval pathways and post-market surveillance systems designed for vulnerable populations. The potential benefits of HFFs in memory disorder management should be weighed against their safety risks through rigorous scientific evaluation to achieve their potential as legitimate therapeutic options while ensuring patient safety and regulatory compliance.

## 6. Clinical Translation of HFFs

The successful translation of multi-target HFFs for memory impairment into market-ready products requires addressing several interconnected challenges that bridge laboratory research and commercial application. As memory-related disorders increase globally, developing accessible, evidence-based cognitive health solutions has become increasingly critical.

Key translation challenges and solutions are as follows.

(1) Formulation Design and Bioavailability Enhancement: Unlike single-compound therapeutics, HFFs contain complex mixtures of bioactive compounds (polyphenols, alkaloids, flavonoids) with varying physicochemical properties and inherently poor bioavailability. To overcome these limitations, advanced delivery systems have been developed including nano-emulsification, liposomal encapsulation, and microencapsulation technologies. The approaches significantly enhance gastrointestinal stability and enable targeted delivery of memory health compounds such as curcumin, ginsenosides, and flavonoid glycosides. Particularly promising are formulations designed for blood–brain barrier penetration, utilizing lipid-based carriers and surface modifications that facilitate central nervous system access [165]. Additionally, co-administration strategies using bioavailability enhancers (e.g., piperine, phospholipid complexation) and timing optimization relative to meals can further improve compound absorption and therapeutic efficacy.
(2) Standardization and Quality Control: Natural product variability due to cultivation conditions, genotype, harvest timing, and processing methods creates significant batch consistency challenges. Effective quality control solutions require dual approaches combining HPLC fingerprinting with bioassay-based functional verification to ensure both chemical consistency and biological activity. While these rigorous methods can create industrial-scale production bottlenecks, implementing automated analytical systems and establishing standardized operating procedures with qualified suppliers can streamline quality assurance processes without compromising product integrity [166].
(3) Product Stability and Shelf-Life Optimization: Cognitive health compounds are often sensitive to oxidation, hydrolysis, and photodegradation [167]. Comprehensive stability solutions include accelerated and real-time stability testing protocols to identify optimal protective excipients (antioxidants, chelating agents), packaging materials (light-protective, moisture-barrier), and storage conditions. Advanced packaging technologies such as blister packs with desiccants, nitrogen-flushed containers, and temperature-indicating labels ensure efficacy maintenance throughout shelf life—particularly crucial for products targeting aging populations with longer storage periods.
(4) Patient Compliance and Adherence Solutions: Memory disorder patients face unique compliance challenges that significantly impact treatment outcomes. Memory impairment can lead to forgotten doses, confusion about dosing schedules, and difficulty distinguishing between different medications or supplements. Evidence-based formulation strategies to improve compliance include developing once-daily sustained-release formulations, creating distinctive packaging with clear labeling and integrated reminder systems, optimizing palatability through taste-masking technologies and preferred delivery formats (liquids, soft gels, chewable tablets), and incorporating digital adherence monitoring tools. Patient-centered design approaches, including smart packaging with dose tracking, mobile app reminders, and simplified administration protocols, are essential for ensuring therapeutic benefits reach the intended population. Healthcare provider education about compliance monitoring and structured family caregiver involvement protocols becomes crucial for successful long-term treatment outcomes.
(5) Regulatory Compliance and Market Access: Marketing memory support products requires careful navigation between permissible structure-function claims and prohibited disease-prevention claims [168]. Successful regulatory strategies include early engagement with regulatory agencies, robust clinical documentation supporting safety and efficacy claims, and development of clear labeling that communicates benefits within approved frameworks. Digital marketing environments require particular attention to claim substantiation and appropriate targeting, especially for products serving vulnerable populations [169]. International market access demands understanding varying regulatory requirements across jurisdictions and adapting product positioning accordingly while maintaining scientific integrity.

Several HFFs have successfully demonstrated memory function improvement in clinical studies (Table 3), providing valuable precedents for future development programs. Successful clinical translation requires integrated collaboration between neuroscientists, formulation chemists, clinical researchers, regulatory experts, patient advocates, and marketing strategists to transform promising bioactive compounds into safe, effective, and accessible cognitive health products, addressing the growing burden of memory decline and neurodegeneration.

## 7. Next-Generation HFF Formula Development: Testable Hypotheses and Conceptual Frameworks

Developing effective HFFs for memory impairment requires moving beyond traditional supplement approaches toward systems-based therapeutics guided by testable hypotheses and innovative frameworks. We propose three interconnected conceptual frameworks that address major field challenges through specific, testable hypotheses for advancing HFF development as follows: (1) The Precision Biomarker Threshold Framework hypothesizes that HFF efficacy detection is governed by personalized biomarker thresholds that vary predictably based on individual biological profiles. The Biomarker Threshold Theory proposes that HFF effects become clinically detectable only when biomarker changes exceed individual baseline variability by more than 20%, with APOE4 carriers requiring 40% greater biomarker changes than non-carriers to achieve equivalent cognitive improvements [170]. Individuals with high baseline inflammation show biomarker responses 2–3 weeks earlier than those with low inflammation, suggesting personalized monitoring protocols could improve effect detection by 60% [171]. The framework can be validated through prospective cohort studies comparing fixed-threshold versus personalized-threshold biomarker monitoring in HFF interventions. (2) The Multi-Modal Biomarker Synergy Framework hypothesizes that biomarker panels demonstrate exponential rather than additive sensitivity to HFF effects, requiring integrated analysis approaches to detect clinically meaningful changes. Combined inflammation, oxidative stress, and neurotrophic factor panels show synergy coefficients of 1.8–2.4 for effective interventions, while single biomarker approaches fail to detect 40–60% of clinically beneficial HFF interventions [172]. The Biomarker Network Amplification Theory suggests that interconnected biomarker changes create cascading effects that amplify detection sensitivity when analyzed as networks rather than individual markers, enabling the development of AI-powered composite biomarker indices that weight individual markers based on their network connectivity and temporal dynamics. (3) The Temporal Biomarker Cascade Framework hypothesizes that biological systems respond to HFFs in predictable temporal sequences, with peripheral changes preceding central nervous system changes. The Microbiome-Brain Biomarker Bridge Hypothesis proposes that gut microbiome changes precede brain biomarker changes by 4–8 weeks, as microbiome-derived metabolites like butyrate and urolithins cross the blood–brain barrier and modulate neuroinflammation before structural brain changes occur [173]. The temporal cascade could predict cognitive responses with over 80% accuracy and enable the development of early-warning biomarker systems that identify HFF responders within 2–4 weeks rather than waiting 12–24 weeks for cognitive changes.

The frameworks require integration of emerging technologies including single-cell spatial transcriptomics for tissue-specific HFF responses, liquid biopsies for minimally invasive dynamic biomarker monitoring, wearable neurocognitive devices for real-time cognitive assessment, and AI-powered multi-omics integration revealing interconnected pathway modulation involving NF-κB, Nrf2, mitochondrial function, and gut–brain interactions [174,175]. The frameworks directly address three major field challenges: inconsistent HFF validation results through precision threshold-based validation, low biomarker sensitivity through multi-modal synergy approaches, and long validation timelines through temporal cascade biomarkers. The systems-based approach, validated through hypothesis-driven research and longitudinal cohort studies utilizing AI-powered clinical trial simulations, represents a paradigm shift toward personalized nutrition interventions that transform HFF development from empirical testing to precision-guided therapeutics optimized for individual cognitive health profiles.

## 8. Conclusions

The next generation of HFFs represents a paradigm shift toward precision nutritional medicine for cognitive health. By moving beyond the single-nutrient approach to synergistic combinations of complementary bioactive compounds guided by individualized biomarker signatures, HFFs can address the complex heterogeneity of cognitive decline. Success requires establishing an integrated translational pipeline connecting network pharmacology, microbiome modulation strategies, and multi-omics validation with robust clinical trial methodologies [176,177]. The evolution positions evidence-based dietary interventions as essential components of personalized neurological healthcare—not merely as supplements but as targeted, biologically active agents capable of a meaningful clinical impact on memory preservation. Through interdisciplinary collaboration between systems biology, neuroinformatics, nutrition science, and clinical neurology, HFFs can become powerful tools in addressing the growing global challenge of cognitive decline and neurodegenerative disorders.

## Figures and Tables

**Table 1:** Comparison of established clinical biomarkers and emerging modulatable biomarkers for memory impairment.

Category	Established Clinical Biomarkers	Detection Method	Emerging Nutritionally Modulatable Biomarkers	Role in HFF Development
Neuroimaging	Hippocampal atrophy (MRI) Aβ/tau deposition (PET)	Structural/Molecular imaging	Default mode network activity (fMRI)	Early functional changes before structural damage
Cerebrospinal Fluid	Aβ42, p-tau, t-tau (AT(N) system)	Lumbar puncture	Neuroinflammatory cytokines (e.g., IL-1β, TNF-α)	Monitor neuroinflammation response to dietary interventions
Blood-Based	Plasma p-tau, Aβ42/40 ratio Neurofilament light chain	Immunoassays	BDNF, miRNA profiles (e.g., miR-132, miR-146a)	Non-invasive tracking of synaptic plasticity and epigenetic regulation
Cognitive Assessment	MMSE, MoCA, ADAS-Cog	Behavioral tests	Digital biomarkers (wearable devices)	Real-world cognitive monitoring
Gut–Brain Axis	-	-	SCFAs (butyrate), gut permeability (zonulin), microbiota diversity	Key targets for prebiotics/probiotics efficacy validation
Oxidative Stress	CSF F2-isoprostanes	Mass spectrometry	Urinary 8-OHdG, serum MDA	Directly responsive to antioxidant-rich HFFs (e.g., berries, turmeric)
Metabolic Dysfunction	-	-	HOMA-IR, AGEs, carnitine profiles	Link metabolic health to cognitive decline; modulated by polyphenols

**Table 2:** Health Functional Food active components: targets and mechanisms in memory impairment through in vitro, in vivo, and clinical trials.

Health Functional Food	Active Ingredients	Therapeutic Targets	Model Organisms			Ref.
			In vitro	In vivo	Human/review	
Phosphatidylserine	Phosphatidylserine	Akt/PKC signaling activation			human	[143,144]
Fibroin extract	Silk fibroin	ERK/JNK/NF-κB pathway inhibition			human	[142]
*Ginkgo biloba* leaf extract	Flavonoids, terpenoids	Akt/mTOR pathway activation; NF-κB inhibition			human	[125]
EPA and DHA	Omega-3 fatty acids	GPR120/PPARγ pathway modulation			human	[30,31,45]
Green tea extract (L-theanine)	L-theanine	NF-κB pathway inhibition			human	[32]
Ginseng and *Acanthopanax* mixture	Saponins, lignans	NLRP3 inflammasome inhibition	N2a/APP695 cell	APP/PS1 mouse	human	[34,37,38]
*Lycium chinense* extract	Polysaccharides, alkaloids	Wnt/NF-κB pathway modulation; PI3K-AKT-mTOR activation			human	[52,89]
BT-11 (*Polygala tenuifolia*)	Tenuifolin, polygalaxanthone III	AChE inhibition; BDNF/TrkB signaling upregulation			human	[88]
High-temp. fermented green tea	Gallocatechin gallate	PKA/NF-κB/MAPK pathway inhibition			human	[51]
Pomegranate-derived metabolites	Urolithin A (microbial metabolite)	Mitochondrial biogenesis enhancement			human	[117]
*Bifidobacterium breve*	–	Tryptophan metabolism modulation (↑ 5-HTP/5-HT)			human	[110]
*Hericium erinaceus*	Not specified	Redox balance, Nrf2 activation		APP/PS1 mouse		[92]
*Forsythia suspensa*	Not specified	SCFA production, cognitive function		Aβ_25–35_–treated SD-rat		[96]
*Cassiae Semen*	Not specified	SCFA production, cognitive function		Aβ_25–35_–treated SD-rat		[96]
*Polygonatum sibiricum*	Polysaccharides	Gut microbiota modulation, neuroinflammation		5xFAD mice		[106]
*Schisandra chinensis*	Gomisin A, Schisandrin	Neuroinflammation, synaptic function		Aβ_25–35_–treated SD-rat	Review	[82]
*Cornus officinalis*	Polysaccharides, Iridoid glycosides	Synaptic plasticity, BDNF-TrkB signaling		Aβ_25–35_-treated mice		[127]
*Zanthoxylum bungeanum*	Hydroxy-α-sanshool	CREB/BDNF axis		scopolamine-treated mice		[129]
*Rubus idaeus*	Flavonoids	Cholinergic function, oxidative stress		VD (Vascular demented) SD rat	Review	[130]
*Portulaca oleracea*	Purslane amide E	Oxidative stress, neurotoxicity		LPS-treated mice		[119]
*Gardenia jasminoides*	Total flavonoids	Cholinergic system, PERK pathway		VD rat model		[120]
Porcine brain enzyme hydrolysate	Leucine, lysine, phenylalanine, tripeptides, or tetrapeptides	Gut–brain axis, memory function		scopolamine-treated SD-rat		[83]
*Dendrobium nobile*	Alkaloids (DNLA)	Tau hyperphosphorylation, Aβ neurotoxicity			Review	[123]
*Panax notoginseng*	Ginsenosides	PI3K/Akt/mTOR pathway, cholinergic neurotransmission	N2a/APP695 cell			[125]
*Rubus fruticosus*	Extract	Memory deficits in vascular dementia		VD(Vascular demented) SD rat		[130]
Cinnamon	Cinnamaldehyde	Mitochondrial dynamics, Aβ accumulation	THP-1 cell			[121]
Inulin	-	Neurotransmitter production, cognitive function		Aβ_25–35_–treated SD-rat		[95]
Luteolin	-	Insulin resistance, neuroinflammation		Aβ_25–35_–treated SD-rat	Review	[44,47]
Resveratrol	-	SIRT1/PGC-1α pathway, mitochondrial biogenesis		severe acute pancreatitis (SAP) SD-rat		[49]
Ferulic Acid	-	Insulin sensitivity, neuroinflammation		Px and Aβ_25–35_ treated SD-rat		[69]
Berberine	-	Bile acid metabolism, neuroprotection		C57BL/6 mice, Aβ1-42 treated mice		[101,102]
Curcumin	-	Bile acid metabolism, neuroprotection		Aβ1-42-treated mice		[102]
*Akkermansia muciniphila*	-	Gut barrier function, neuroinflammation		APP/PS1 mice		[108]
*Lactobacillus*	-	Dopaminergic signaling, cognitive function	HT22 nerve cell	Tg-APP/PS1 mice		[124]

↑: Increase. Aβ: Amyloid-beta; BDNF: Brain-derived neurotrophic factor; SCFA: Short-chain fatty acids; Nrf2: Nuclear factor erythroid 2-related factor 2; CREB: cAMP response element-binding protein; PERK: Protein kinase R-like endoplasmic reticulum kinase; PI3K: Phosphoinositide 3-kinase; Akt: Protein kinase B; mTOR: Mammalian target of rapamycin; SIRT1: Sirtuin 1; PGC-1α: Peroxisome proliferator-activated receptor gamma coactivator 1-alpha; TrkB: Tropomyosin receptor kinase B; DNLA: Dendrobium nobile alkaloids.

**Table 3:** Health functional ingredients claiming memory health in the literature: Active Compounds, Dosages, Targeted Pathways, and Modulated Biomarkers in in vitro, in vivo, and clinical trials.

Health Functional Food	Dosage (mg/day)	Active Compounds (s)	Targeted Pathways	Modulated Biomarkers	Ref.
Phosphatidylserine	300~800	Phosphatidylserine	Akt, protein kinase C (PKC), and Raf-1 signaling activation	Phosphatidylserine	[150,151,166,167]
Fibroin extract	200~400	Silk fibroin	ERK signaling activation, JNK signaling pathway activation, NF-κB signaling pathway activation	IL-1β ↓, IL-6 ↓, TNF-α ↓, brain ACh level ↑	[130,147]
*Eriobotrya folium* extract	1500	Flavonoids, quercetin, triterpenoid acids, sesquiterpene glycosides	iNOS expression inhibition, COX-2 expression inhibition, NF-κB binding activity inhibition	NO ↓, PGE2 ↓, iNOS ↓, COX-2 ↓, NF-κB ↓, MAPK phosphorylation ↓	[28,29]
BT-11 (*Polygala tenuifolia* Willd.) extract	300	*Tenuifolin, senegenin*, polygalacic acid, polygalaxanthone III, 3.6′-disinapoyl sucrose, polygalacic acid	Acetylcholine enzyme inhibition, ERK, cAMP, NF-κB, BDNF/TrkB signaling pathway	TNF-α ↓, IL-1β ↓, IL-6 ↓, IFN-γ ↓, LPS ↓, SOD ↑, GSH level ↑	[89]
EPA and DHA	900~2000	Omega-3 fatty acid	GPR120/PPARγ pathway inhibition, IFN-γ secretion inhibition, WAT NLRP3 inflammasome/IL-1β pathway upregulation	TNF-α ↓, IFN-γ ↓	[30,31,45]
Green tea extract theanine compounds	1680	L-theanine	NF-κB pathway inhibition	IL-23 ↓, IL-1β ↓, TNF-α ↓, COX-2 ↓, IL-17A ↓	[32]
High temperature treated green tea extract	900	Gallocatechin gallate, catechin	PKA Pathway modulation, NF-κB and MAPK pathways inhibition	Improve glucose tolerance, insulin sensitivity	[51]
Ginkgo leaf extract	120	Flavonoids(quercetin, kaempferol, isorhamnetin), Terpenoids(ginkgolides, bilobalide)	Activates the Akt/mTOR pathway, PI3K/AKT signaling pathway upregulation, modulates AMPK-mTOR Pathway, and NF-κB pathway inhibition	IL-1β ↓, TNF-α ↓, IL-6 ↓, SIRT-1 expression ↑	[113,128]
Ginseng and *Acanthopanax Koreanum* mixture	5200	Proanthocyanidin, triterpenoid saponins, lignans, coumarins, flavones, phenolic compounds, acankoreosides	NF-κB pathway inhibition, NLRP3, and dopaminergic pathways, tumor necrosis factor alpha-α inhibition	Antioxidant activities (ABTS, FRAP, reducing power, ORAC), NLRP3 inflammasome ↓	[34,37,38,92]
*Lycium chinense* extract	1425	Polysaccharides, alkaloids, flavonoids	WNT pathway activation, aberrant NF-κB activation inhibition, PI3K-AKT-mTOR signaling activation, BCL2-Associated X (Bax)/B-cell lymphoma-2 (Bcl-2) downregulation	BDNF expression ↑, amyloid-beta (Aβ) deposits ↓, tau phosphorylation ↓, TNF-α ↓, iNOS ↓, IL-1β ↓, COX-2 ↓	[52,90]

↑, Increase; ↓, Decrease. ACh, acetylcholine; Aβ, amyloid-beta; ABTS, 2,2′-azino-bis(3-ethylbenzothiazoline-6-sulfonic acid); Akt, protein kinase B; AMPK, AMP-activated protein kinase; BDNF, brain-derived neurotrophic factor; Bax, BCL2-associated X protein; Bcl-2, B-cell lymphoma 2; cAMP, cyclic adenosine monophosphate; COX-2, cyclooxygenase-2; ERK, extracellular signal-regulated kinase; FRAP, ferric reducing ability of plasma; GPR120, G-protein coupled receptor 120; GSH, glutathione; IFN-γ, interferon-gamma; IL, interleukin; iNOS, inducible nitric oxide synthase; JNK, c-Jun N-terminal kinase; LPS, lipopolysaccharide; MAPK, mitogen-activated protein kinase; mTOR, mechanistic target of rapamycin; NF-κB, nuclear factor kappa-light-chain-enhancer of activated B cells; NLRP3, NOD-like receptor family pyrin domain containing 3; NO, nitric oxide; ORAC, oxygen radical absorbance capacity; PGE2, prostaglandin E2; PI3K, phosphoinositide 3-kinase; PKA, protein kinase A; PPARγ, peroxisome proliferator-activated receptor gamma; SIRT-1, sirtuin 1; SOD, superoxide dismutase; TNF-α, tumor necrosis factor alpha; TrkB, tropomyosin receptor kinase B; WAT, white adipose tissue; WNT, wingless/integrated signaling pathway.

## Data Availability

Data available on request from the authors.
